# Extreme value theory inspires explainable machine learning approach for seizure detection

**DOI:** 10.1038/s41598-022-15675-9

**Published:** 2022-07-06

**Authors:** Oleg E. Karpov, Vadim V. Grubov, Vladimir A. Maksimenko, Semen A. Kurkin, Nikita M. Smirnov, Nikita P. Utyashev, Denis A. Andrikov, Natalia N. Shusharina, Alexander E. Hramov

**Affiliations:** 1 National Medical and Surgical Center named after N. I. Pirogov, Ministry of Healthcare of the Russian Federation, Moscow, Russia; 2 Neuroscience and Cognitive Technology Laboratory, Innopolis University, Kazan, Russia; 3 Baltic Center for Artificial Intelligence and Neurotechnology, Immanuel Kant Baltic Federal University, Kaliningrad, Russia; 4 Research and Production Company “Immersmed”, Moscow, Russia; 5 Department of Theoretical Cybernetics, Saint Petersburg State University, St. Petersburg, Russia

**Keywords:** Diseases of the nervous system, Epilepsy, Scientific data, Information technology, Diagnostic markers, Nonlinear phenomena, Phase transitions and critical phenomena

## Abstract

Epilepsy is one of the brightest manifestations of extreme behavior in living systems. Extreme epileptic events are seizures, that arise suddenly and unpredictably. Usually, treatment strategies start by analyzing brain activity during the seizures revealing their type and onset mechanisms. This approach requires collecting data for a representative number of events which is only possible during the continuous EEG monitoring over several days. A big part of the further analysis is searching for seizures on these recordings. An experienced medical specialist spends hours checking the data of a single patient and needs assistance from the automative systems for seizure detection. Machine learning methods typically address this issue in a supervised fashion and exhibit a lack of generalization. The extreme value theory allows addressing this issue with the unsupervised machine learning methods of outlier detection. Here, we make the first step toward using this approach for the seizure detection. Based on our recent work, we specified the EEG features showing extreme behavior during seizures and loaded them to the one-class SVM, a popular outlier detection algorithm. Testing the proposed approach on 83 patients, we reported 77% sensitivity and 12% precision. In 60 patients, sensitivity was 100%. In the rest 23 subjects, we observed deviations from the extreme behavior. The one-class SVM used a single subject’s data for training; therefore, it was stable against between-subject variability. Our results demonstrate an effective convergence between the extreme value theory, a physical concept, and the outlier detection algorithms, a machine learning concept, toward solving the meaningful task of medicine.

## Introduction

Extreme events are rare abnormal deviations of the system’s behavior from a typical state^1–3^. From the statistical perspective, extreme events occur in the tails of a probability distribution. In the absence of extreme events, the probability distribution has typically a gaussian shape with exponential tails. In the presence of extreme events, these tails become heavy and start following the power law with a fixed power $\sim p^{-\alpha }$, $\alpha >0$. Sometimes, extreme events (severe rains, economic crises, tsunamis) occur in real-world systems and harm human life. Therefore, an important task is understanding the developing mechanisms of these events to predict their occurrence in the future^4–7^.

On the time series, extreme events correspond to outliers. Outlier is a general term in statistics and data science that characterizes data points that differ significantly from other observations. Outliers can occur by chance in any data, but they also indicate that a system has a heavy-tailed distribution. Outliers reduce the statistical explanation abilities for traditional data-driven modeling methods. Therefore, an essential part of data analysis is searching for outliers and removing them from the data^8^.

Recently scientists started considering spontaneous synchronous activity in neuronal populations as extreme events, referring the epileptic seizures as their brightest example^3,9–11^. In the previous studies, we found the signs of extreme behavior on epileptic electroencephalograms (EEG). First, we revealed that the onsets of seizures in rats demonstrate on-off intermittency dynamics, similar to extreme events^12–14^. Then, we reported heavy tails on the probability distributions of the spectral power of epileptic EEG^15^. Finally, we considered EEG of the patients with epilepsy and revealed that the probability distribution of the spectral power in the 1–5 Hz frequency range also has a heavy tail manifesting extreme behavior^16^. Thus, epileptic seizures in humans are extreme events that cause outliers on the time series of the 1–5 Hz EEG spectral power. The connection between extreme events and outliers opens the way for using a vast repertory of outlier detection techniques for seizure detection.

According to the International League Against Epilepsy and the International Federation of Clinical Neurophysiology, there is a need for automated seizure detection to decrease morbidity and mortality associated with seizures and for objective seizure identification and quantification^17^. The authors concentrated on the wearable devices for seizure detection on an outpatient basis. In contrast, we focus on seizure identification in hospital settings, which also plays a crucial role in epilepsy diagnostics and treatment. Usually, treatment strategies start by analyzing brain activity during seizures revealing their type and onset mechanisms. This approach requires collecting data for a representative number of events which is only possible during the continuous EEG monitoring over several days. Based on 248 patients, Friedman and Hirsch concluded that it is common to require more than three days in an epilepsy monitoring unit to record and diagnose the nature of paroxysmal episodes and not rare to require more than a week^18^. A big part of the further analysis is searching for seizures on these recordings. An experienced specialist spends hours reviewing the data of a single patient and needs assistance from automated systems for seizure detection. While the fully automated diagnostic remains a matter of discussion, the working solution is partial automation, known as the Clinical decision support system (CDSS)^19^. In CDSS, the computer analyses data and provides recommendations, and the medical expert makes the final decision.

An optimistic approach to seizure detection is machine learning (ML)^20^. For the ML, seizure detection comes down to classifying EEG data in two classes, seizures (class 1) and non-seizures (class 2)^21,22^ . The classification requires ML algorithms to be trained on the training data. This approach, a supervised classification, suffers from the class imbalance and overfitting. The class imbalance comes from the rare nature of seizures and requires artificially balancing the number of the class 1 and the class 2 examples in the training data. A promising way to manage the imbalance is constructing feature space resulting in the long distance between classes. The modern deep learning algorithms handle it well but suffer from overfitting. The overfitting implies that the algorithm performs satisfactorily on the training data but fails to operate with data of the other patients. Addressing this issue relies on selecting a subspace of features containing the biomarkers of seizures common for the most patients. These reasonings lead us to the problem of the features interpretability which often occurs in machine learning.

We propose using the outlier detection ML technique, one-class SVM, to overcome these issues. First, this method incorporates the rare nature of seizures, hence handling the class imbalance problem. Second, it is unsupervised and may train and perform on the data of a single patient. Finally, we will feed the algorithm with an interpretable feature (EEG power in the 1–5 Hz frequency range) which demonstrates extreme behavior during seizures. We suppose that interpretability gives us a prior knowledge about the ability of an algorithm to handle the data of this patient, hence assessing its credibility.

## Methods

### Dataset

The experimental dataset under study was provided by the National Medical and Surgical Center named after N. I. Pirogov of the Russian Healthcare Ministry (Moscow, Russia). All medical procedures were held in the Center following the Helsinki Declaration and the Center’s medical regulations and were approved by the local ethics committee. All patients provided written informed consent before participation. The dataset includes anonymized long-term monitoring data of patients in the Department of Neurology and Clinical Neurophysiology between 2017 and 2019. The data were collected during routine medical procedures aimed at registration of epileptic activity and verification of epileptogenic zones for further clinical treatment. Continuous EEG and video monitoring during everyday activity including sleep and wakefulness were performed for these patients. During the monitoring, patients kept a regular daily routine with occasional physiological trials (such as photostimulation and hyperventilation) that are standard for this type of research^23,24^. The length of recording varied from 8 to 84 h according to the patient’s condition and the number of epileptiform activity episodes needed for the proper diagnosis. Each patient had from one to five epileptic seizures during the time of the monitoring. None of the seizures was triggered by photostimulation or hyperventilation, i.e., all epileptic seizures were spontaneous. In this work, we use the dataset which contains the data of 83 patients diagnosed pathologically with focal epilepsy. Epileptic focuses were found in frontal, temporal, or parietal areas of left, right, or both hemispheres, so there was no homogeneity in the dataset in terms of the epileptic focus localization.

### Data acquisition and preprocessing

“Micromed” encephalograph (Micromed S.p.A., Italy) was used for EEG recording. EEG signals were recorded with 25 channels according to the international “10–20” system with a ground electrode placed on the forehead and reference electrodes placed at the ears. EEG signals were recorded with a sampling rate of 128 Hz. The video monitoring system was used to monitor patients’ states for easier data analysis and segmentation. Experimental EEG data and video recordings were examined and deciphered by an experienced neurophysiologist. This analysis resulted in the marking that includes information on episodes of epileptic seizures and physiological tests.

EEG signals are known to be highly susceptible to the influence of various external and internal noises, especially during prolonged recording^25^. In clinical monitoring, external noises usually come from hardware-related problems (poor contact of EEG electrodes, loose wires), power network, cellphone calls, etc. Internal noises (or physiological artifacts) originate from physiological processes such as heartbeat, blinking, or breathing^26^. The basic way to deal with noises and artifacts on EEG is the data filtration: a high-pass filter is used to restrain the low-frequency components (stray effects, cardiac rhythm, breathing artifacts), while a low-pass filter deals with the high-frequency activity, commonly associated with the muscle artifacts. In our study, we used the band-pass filter with the cutoff frequencies of 1 Hz and 60 Hz and the 50-Hz notch filter.

We considered the frequency band 1–30 Hz, which is commonly regarded as an effective frequency range of epileptic EEG. However, some undesired activity (e.g., eye-movement artifacts) can interfere with the EEG activity in this range. We used the standard procedure based on an independent component analysis (ICA) to remove these artifacts^27^.

### Time-frequency analysis

We performed a time-frequency analysis of EEG signals using continuous wavelet transform (CWT) with Morlet mother wavelet function^28^. CWT has proved itself as a powerful instrument in the analysis of complex nonstationary signals in systems of different nature, including biological ones^29^. We considered wavelet power (WP):

1 $$\begin{aligned} W_n(f,t)=|w_n(f,t)|, \end{aligned}$$

where *n* = 1, 2...*N* is the number of EEG channel ($N = 25$ for the considered dataset), *f* and *t* are the frequency and time point, $w_n(f,t)$ are the coefficients of CWT. WP is one of the common CWT-based characteristics to describe the time-frequency structure of a signal^30^.

CWT can be demanding in terms of computational costs: application of CWT in the frequency range of interest to several day-long multichannel EEG data with even low sampling rate will generate an immense amount of WP data. Such a big dataset is difficult to calculate, store, and analyze, so some approaches to reduce data complexity are required.

The first step includes averaging WP over the frequency band of interest. Initially, WP was calculated in the 1–30 Hz frequency range as it is acceptable for both normal and epileptic EEG^31^. Earlier in our paper^15^, we applied extreme event theory to analyze electrocorticogram (ECoG) recordings of WAG/Rij rats with a genetic predisposition to the absence epilepsy. We reported that the absence seizures induced a drastic increase of WP in the frequency range of 6–8 Hz, and WP distribution is best fitted with the Weibull function after averaging WP over the 6–8 Hz range. The WP time series demonstrated extreme events-related properties in this frequency range, while there were no manifestations of the extreme behavior for other frequencies. We conducted similar research in our paper^16^ for epilepsy in human patients and showed that WP averaged over the frequency range of 2–5 Hz demonstrates extreme-like behavior during epileptic seizures. So, we choose the $F \in [2;5]$ Hz range as the frequency band of interest in the present study.

The second step includes averaging WP over the 25 EEG channels. This step can be explained by the features of epilepsy—in the case of generalized seizures, activity arises suddenly all over the brain, and all EEG signals are highly correlated^32^. In focal seizures, there are only a few EEG channels near the focus that demonstrate pronounced epileptic activity, however, these channels stand out in terms of EEG signal amplitude and frequency structure, so even after averaging over the channels WPs for normal and pathological activity differ drastically.

Thus, we calculated averaged over the frequency band $F \in [2;5]$ Hz and $N=25$ EEG channels WP (AWP) as:

2 $$\begin{aligned} E(t)=\frac{1}{N}\sum _{n=1}^{N} \frac{1}{\Delta F} \int \limits _{f \in F} W_n(f,t)df, \end{aligned}$$

where $\Delta F=3$ Hz is the width of the frequency band $F \in [2;5]$ Hz.

We applied “downsampling” of AWP to decrease the complexity of the data additionally. We divided each EEG recording into 60-s intervals $T_m$, where $m = 1, 2...M$, $M =L//60$, *L*—the length of EEG recording in seconds, “//” stands for integer division. The choice of such interval length is justified by the average duration of an epileptic seizure— from 30 to 120 s^33^. AWP values were calculated for each time interval *m* and averaged over the whole length of the interval to obtain downsampled AWP (DAWP):

3 $$\begin{aligned} e_m=\frac{1}{\Delta T} \int _{t \in T_m} E(t)dt, \end{aligned}$$

where $\Delta T$ is the length of each interval $T_m$ ($\Delta T = 60$ s).

### Probability density function

To construct a probability density function (PDF), we normalized *E*(*t*) and $e_m$ by corresponding global minimum as: $E(t)^{norm} = E(t) - E_{min}$, $e_m^{norm} = e_m - e_{min}$. For $E(t)^{norm}$, we additionally extracted all local maxima. According to the Fisher–Tippett–Gnedenko theorem^34^, if a Gumbel, Frechet, or Weibull distribution describes the obtained PDF, then the process is characterized by extreme event properties. Our previous studies on epilepsy in the WAG/Rij rats^15^ and human patients^16^ suggest that the Weibull distribution is more suitable for this task. Thus, PDFs for EEG data were fitted by the Weibull distribution in the present work^35^:

4 $$\begin{aligned} f(x,c)=c\left( \frac{x-l}{s} \right) ^{c-1}\exp \left( -\left( \frac{x-l}{s} \right) ^c \right) , \end{aligned}$$

where *c* is the shape parameter of the Weibull law ($x>0$, $c>0$), *l* is the localization parameter that shifts the distribution with fixed shape parameter *c* across the axis of WP, *s* is the scale parameter responsible for the expansion/compression of the initial distribution.

**Figure 1:**
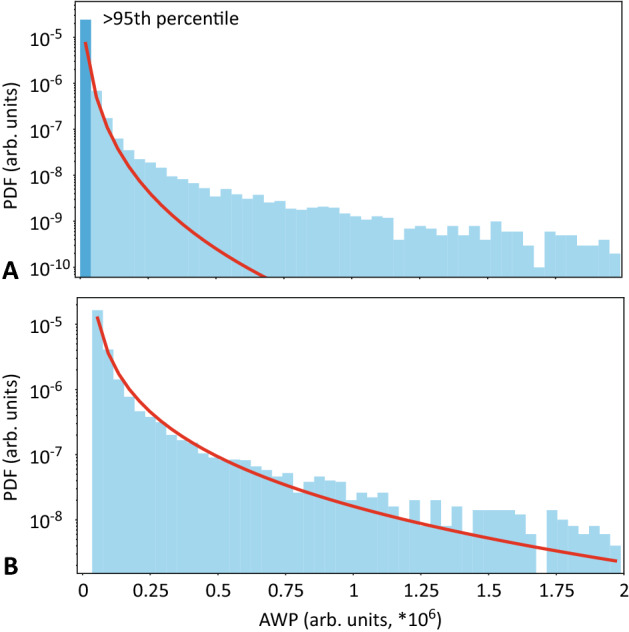
PDF of $E(t)^{norm}$ for one subject for: (**A**) the whole dataset and (**B**) above 95th percentile subset; dark blue histogram shows below 95th percentile subset, blue histogram—above 95th percentile subset; the Weibull approximations are shown as red lines.

In our previous papers^15,16^, we have shown that experimental PDFs for normal and epileptic activity are fitted by Weibull distributions with drastically different parameters, so it can be problematic to properly fit the whole dataset with a single Weibull distribution. Figure 1 illustrates this issue, demonstrating the PDF of $E(t)^{norm}$ for one subject as the blue histogram and the fitted Weibull distribution as the red line. It is clear from Fig. 1A that the tail of the Weibull distribution, in this case, is poorly fitted. In the present study, we are mainly interested in epileptic extreme events that would obviously lie within the tail of the Weibull distribution. With this in mind, we applied additional preprocessing prior to fitting experimental data with Weibull distribution. We have shown in Refs.^15,36^ that the cross point between normal and extreme behavior distributions can be found using the Pickands-Balkema-de Haan theorem and assessed empirically. In the present study, we proposed another approach for separating “normal” and “extreme” distributions. We calculated the 95th percentile of the analyzed data and divided the whole dataset into two subsets: above the 95th percentile (shown as a blue histogram in Fig. 1A) and below the 95th percentile (shown as a dark blue in Fig. 1A). We suggested that 5% of data (above the 95th percentile) should mainly consist of extreme events, while the rest $95\%$ (below the 95th percentile) should contain the normal data. We selected the above 95th percentile subset and fitted it with the Weibull distribution; Fig. 1B shows that the fitting becomes more accurate.

According to Ref.^15^, we used the chi-square goodness-of-fit test to be sure that the Weibull distribution is appropriate to fit the studied experimental data. The chi-square goodness-of-fit test determines if a data sample comes from a specified probability distribution, with parameters estimated from the data. The test groups the data into bins, calculating the observed and expected counts for those bins and computing the chi-square test statistic.

5 $$\begin{aligned} \chi ^2=\sum _{h=1}^H (O_h-E_h)^2/E_h, \end{aligned}$$

where $O_h$ are the observed counts and $E_h$ are the expected counts based on the hypothesized distribution, $h=1,2...H$ is the number of bins in PDF distribution.

To fit the experimental PDFs with the Weibull distribution and perform the goodness-of-fit test, we used NumPy and SciPy libraries from Python, namely, statistical functions of the special module (*scipy.stats*). The function *stats.weibull_min.fit* was applied to PDF data—it returns maximum likelihood estimations for *c*, *l*, and *s* parameters of the Weibull distribution for the data (PDF). The function *numpy.percentile* was used to calculate the 95th percentile. The function *stats.chisquare* was used to calculate the one-way chi-square test.

### Machine-learning algorithm

One of the most common unsupervised ML algorithms is one-class SVM^37–39^. In this work, we addressed the aforementioned issues by proposing a new approach that uses a “non-black-box” unsupervised ML algorithm, one-class SVM, fed with the features extracted following the fundamental knowledge about human seizures. We used SVM with a kernel in the form of a radial basis function and the standardized predictor. The Iterative Single Data Algorithm (ISDA)^40^ was used for the classifier optimization.

**Figure 2:**
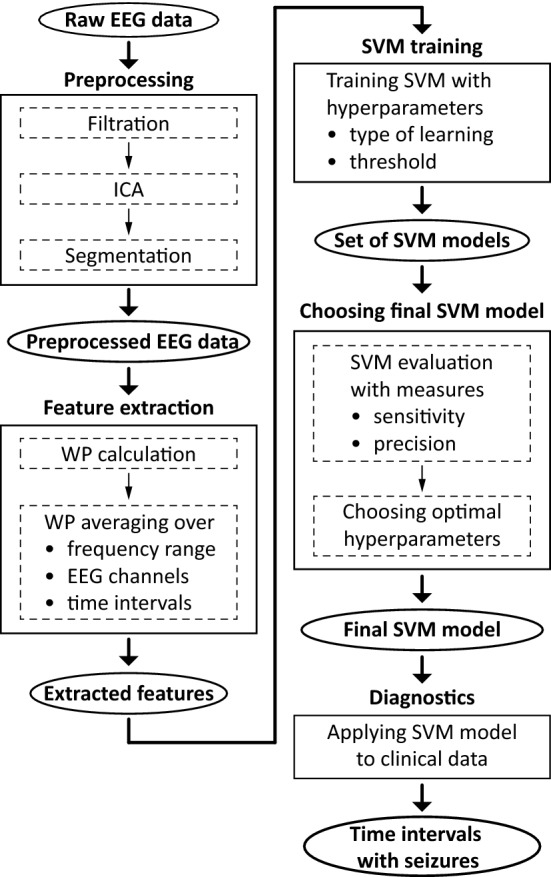
The framework of the ML algorithm. Rectangle frames depict steps of data analysis, oval frames correspond to the input/output data for each step.

**Figure 3:**
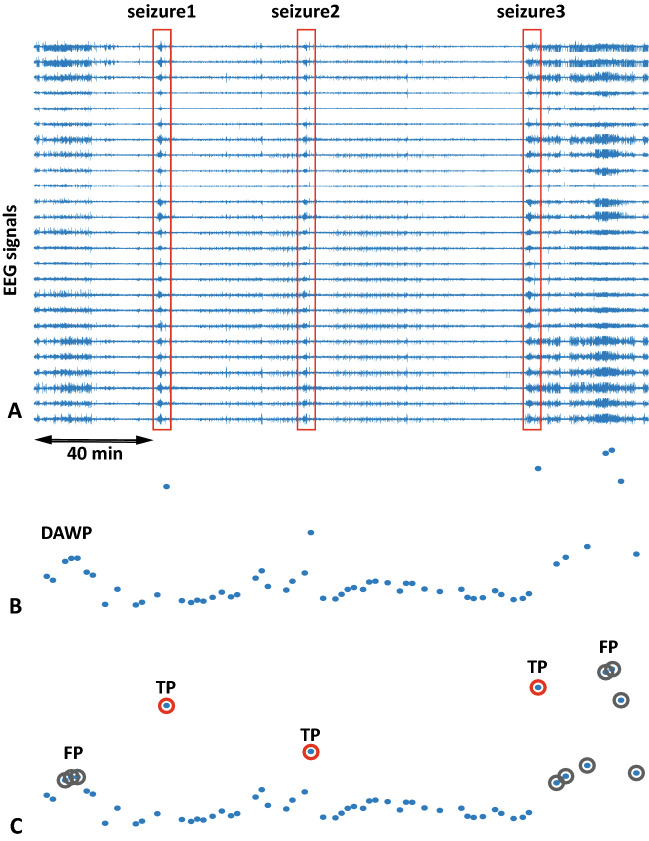
Example of data obtained at various steps of the algorithm: (**A**) EEG data (25 channels) with 3 epileptic seizures (shown as red frames); (**B**) result of WP averaging over the 2–5 Hz frequency range, 25 channels, 60-s time intervals; (**C**) result of ML training and testing with a number of correctly (TP) and falsely (FP) detected events (shown as red and grey circles correspondingly).

The overall framework of the ML algorithm is illustrated in Fig. 2, while Fig. 3 depicts examples of data obtained from certain steps of the algorithm. The first step of the algorithm took raw EEG data as the input and performed preprocessing. This included filtration and ICA-based artifact removal as well as EEG data segmentation into a number of 60-s intervals—the result of preprocessing step is shown in Fig. 3A. Then the preprocessed data served as the input for the feature extraction step. The features for the SVM algorithm were extracted according to the results of the time-frequency analysis of human epileptic EEG. During the feature extraction procedure based on expert-level domain knowledge the following actions were performed:

1. CWT was applied to EEG data intervals and WP was computed in the frequency range of 1–30 Hz;
2. WP was averaged over:
  - 2–5 Hz frequency band of interest;
  - 25 EEG channels;
  - length of each time interval (60 s);

Figure 3B illustrates the result of feature extraction.

Obtained features were used as the input for the SVM training step. At this step, we trained multiple SVM models with different hyperparameters. There were two hyperparameters of the algorithm that might affect its performance:

- *Type of learning* is a strategy used in learning and approbation of the algorithm. We considered two approaches:
  - *k*-*fold cross-validation (CV):* the dataset is randomly permuted and split up into *k* groups or folds, then, for each *k*, the *k*th group is taken as a test data set, while the remaining $k-1$ groups comprise the training data set^41^. In our case, we used data of one subject as a dataset and chose $k=10$.
  - *“Leave-one-out cross-validation” (LOO):* a configuration of *k*-fold cross-validation where *k* is set to the number of examples in the dataset^42^. In our case, we set *k* to the number of subjects, i.e. we used data of all subjects except one in learning and then verified the algorithm on the data of the excluded subject.
- *The threshold* is the expected proportion of outliers in training data. SVM algorithm trains the bias term such that 100$\times$ Threshold% of the observations in the training data have negative scores and will be considered as “outliers”. The minimum achievable threshold value is determined by dataset properties and SVM classifier parameters. Actually, by applying the threshold, we consider only some fraction of these events with the highest AWP values. We assume that outliers corresponding to extreme events—epileptic seizures—should be the ones with the highest values of AWP. Thus, with the application of the threshold, we aim to separate epileptic seizure outliers from others caused by noises and artifacts. We tested eight values of the threshold: 10, 5, 2.5, 1, 0.5, 0.25, 0.1, 0.05%.

After this step, we obtained a set of SVM models with different hyperparameters. We used this set of SVM models as the input for the next step, where we aimed to find the most efficient model and corresponding optimal hyperparameters. To evaluate the efficiency of the ML algorithm, we used several characteristics derived from a confusion matrix^43^:

- *True positive (TP):* correctly identified events, i.e. identified as epileptic activity by both ML method and expert.
- *False positive (FP):* falsely identified events, i.e. identified as epileptic activity by the ML method but not by an expert.
- *False negative (FN):* missed events, i.e. identified as epileptic activity by an expert but not by the ML method.

Each event was represented by one or several 60-s intervals $T_m$ (when the algorithm identifies/misses several consecutive intervals, they are treated as one event). Examples of these events are shown in Fig. 3C.

Using these characteristics, we evaluate the efficiency of our algorithm in terms of two measures: sensitivity and precision. In diagnostics, sensitivity is a measure of how well a test can identify true positives, or, in our case, the probability of a positive test given that the patient has epilepsy. Precision is the probability that subjects with a positive screening test truly have the disease. To reflect sensitivity, we calculated the True Positive Rate (*TPR*) as:

6 $$\begin{aligned} TPR = \frac{TP}{TP+FN} \times 100\%, \end{aligned}$$

Precision or the Positive Predictive Value (*PPV*) is commonly defined as:

7 $$\begin{aligned} PPV = \frac{TP}{TP+FP} \times 100\%. \end{aligned}$$

Dependence of *TPR* and *PPV* on threshold value is usually quite simple for classifiers: *TPR* decreases and *PPV* increases with the decrease of the threshold value. Thus, the choice of the optimal threshold value is dictated by balancing between appropriate *TPR* and *PPV*. However, the effect of the type of learning is not so obvious and requires statistical analysis.

We performed the group-level statistics for *TPR* and *PPV*. For statistical analysis, we used Wilcoxon signed-rank test since the samples’ distribution is abnormal. After that, Holm correction was applied. Normality was tested via the Kolmogorov-Smirnov test. We performed a statistical analysis using SciPy and Statsmodels packages for Python.

After finding the optimal SVM model, we used it to analyze the clinical data and obtain the final results—the marking with time intervals that correspond to epileptic seizures (see Fig. 3C).

## Results

**Figure 4:**
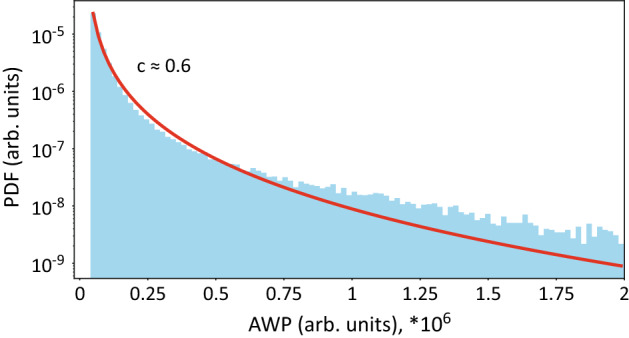
PDF of $E(t)^{norm}$ for all subjects (histogram) and fitted Weibull distribution (red line).

To verify the presence of extreme behavior in the analyzed data, we fitted AWP data of all 83 subjects with the Weibull distribution, as we described in the Methods section. Figure 4 shows the PDF of $E(t)^{norm}$ for all subjects as the blue histogram and fitted Weibull distribution as the red line. According to the Pickands–Balkema–de Haan theorem^44,45^, the elongated tail of experimental distribution fitted by the “heavy-tailed” Weibull distribution (shape parameter $c \sim 0.6$) is the sign of extreme behavior (see Fig. 4). This finding also agrees quite well with our other paper^15^. We can conclude that our data demonstrates extreme behavior and thus can be subjected to techniques aimed at detection of “outliers”, such as SVM.

We applied the developed SVM to the data and calculated *TPR* and *PPV*. At first, we calculated *TPR* and *PPV* for different values of threshold. Results are shown in Table 1: we calculated *TPR* and *PPV* values averaged over all 83 subjects for each value of threshold (10, 5, 2.5, 1, 0.5, 0.25, 0.1, 0.05%) and each type of learning (CV and LOO). We can see that for CV *TPR* decreases and *PPV* increases with the decrease of the threshold value, as expected. However, an increase of *PPV* past the threshold value of 0.5% becomes less noticeable while *TPR* is still high. For LOO, *TPR* also decreases with the decrease of the threshold value, but *TPR* values are generally lower than in the case of CV. Thus, we focused on *TPR* and *PPV* for CV and selected the threshold value of 0.5% to be used in the SVM classifier.

**Table 1:** Results of data analysis with SVM classifier: threshold influence.

Threshold, %	TPR mean, %		PPV mean, %	
	CV	LOO	CV	LOO
10	93.53	85.10	4.79	10.22
5	85.66	73.09	7.05	13.66
2.5	81.72	54.08	10.34	12.16
1	79.08	44.21	12.22	10.77
0.5	76.98	39.96	12.70	9.30
0.25	76.98	39.56	13.07	9.00
0.1	76.67	39.56	13.09	9.00
0.05	76.67	39.56	13.09	9.00

As we mentioned above, mean *TPR* is noticeably higher for CV than for LOO, thus comparison of these two types of learning via statistical test is required for better representation. With chosen threshold, we calculated *TPR* and *PPV* values for two types of learning—the results are shown in Table 2. We found a significant main effect of type of learning on *TPR* but surprisingly not on *PPV*. Learning and testing with CV resulted in $TPR = 76.97 \pm 4.40$ (mean ± standard error (SE)) that was significantly ($p = 3.18 \times 10^{-7}$) higher than for LOO—$TPR=39.96 \pm 5.20$. However, there was no significant effect ($p=0.12$) for *PPV*: $PPV=12.70 \pm 1.47$ for CV vs $PPV=9.30 \pm 1.72$ for LOO. Thus, we chose CV as a more appropriate type of learning and considered these *TPR* and *PPV* values as the final result of the SVM algorithm. The value of *TPR* is acceptable for a simple unsupervised classifier, and it possibly can be improved by further tuning of classifier’s hyperparameters. The value of *PPV* is comparatively low, but we should take into account the heavy skewness of EEG data and the considerable length of recordings. In the end, the results of the proposed classifier are not outstanding and cannot compete with some supervised classifiers in terms of sensitivity or precision. However, the primary goal of this SVM classifier was to demonstrate that the link between epilepsy and extreme behavior opens up the opportunity for novel approaches in EEG data analysis.

**Table 2:**
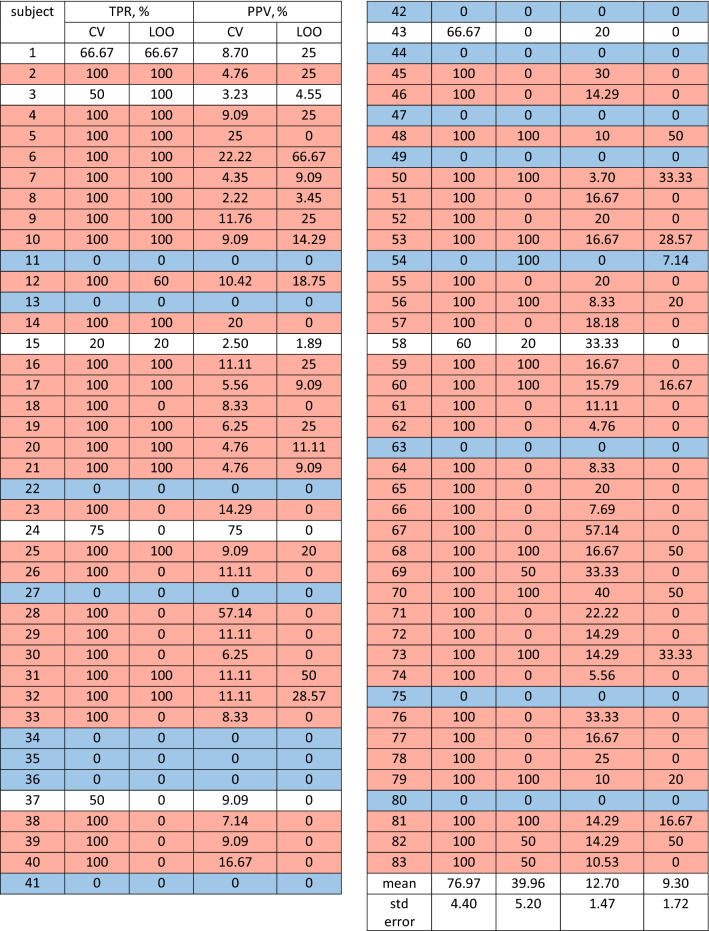
Results of data analysis with SVM classifier: type of learning influence.

*TPR* in most cases (76 out of 83 subjects) has one of the two opposite values: 100% (60 subjects) or 0% (16 subjects). On one hand, this may be caused by a small number of seizures in each subject, usually, only one, so $TPR=100\%$ when this one seizure is detected and $TPR=0\%$ when it is not detected. On the other hand, there are some instances where multiple seizures were all detected or all missed—this may suggest some unexpected features in the data that make difference. To study this possibility, we formed and analyzed two datasets: subjects with $100\%\, TPR$ (shown as red in Table 2) and subjects with $0\% \,TPR$ (shown as blue).

**Figure 5:**
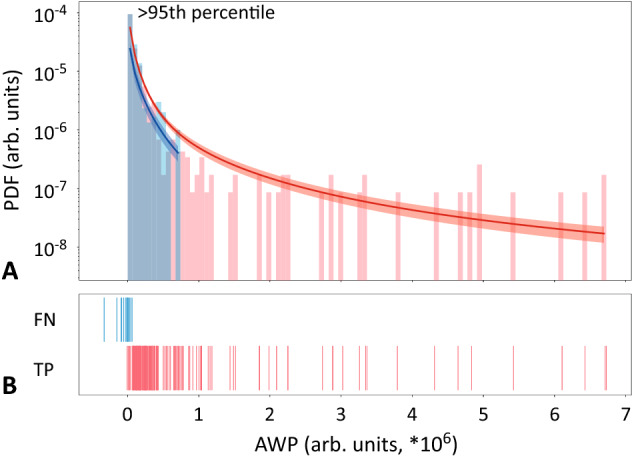
(**A**) PDF of $e_m^{norm}$ for $100\% TPR$ (shown as red histogram) and $0\%\, TPR$ (blue histogram) and group Weibull approximation (mean ± SE) for $100\%\, TPR$ dataset (red) and $0\% TPR$ dataset (blue); (**B**) marking for TPs (red) and FNs (blue).

We hypothesized that extreme behavior somehow differs between these two datasets. To check the hypothesis, we performed an analysis similar to that presented in Fig. 4, but this time we considered DAWP. We constructed PDF individually for each subject and fitted it with the Weibull distribution. We analyzed Weibull distributions for two datasets separately and calculated the mean ± SE for each dataset. Figure 5A demonstrates PDFs for $100\% TPR$ (shown as red histogram) and $0\% \,TPR$ (blue histogram). Fitted Weibull distributions are shown as solid lines and shaded areas (mean ± SE): red for $100\% \,TPR$ and blue for $0\% \,TPR$. Figure 5B shows marking with TPs (red) and FNs (blue) that were obtained during classification. PDF for $100\%\, TPR$ group possesses a more evident “heavy tail”, and we speculate that it corresponds to more pronounced extreme behavior. To check this, we performed the statistical analysis and compared characteristics of fitted Weibull distributions for $100\% \,TPR$ and $0\% \,TPR$ groups. The analysis showed that shape parameter *c* was significantly ($p<0.005$) higher for $100\% \,TPR$ dataset, which explains the “extreme” tail of the Weibull distribution. Additionally, one can see that TPs are distributed in the tail, while many FNs lie in the $<0$ area, which suggests that they did not make to the 95*th* percentile.

## Discussion

We propose using the outlier detection ML technique, one-class SVM, to detect epileptic seizures. Testing our approach on 83 patients, we reported 77% sensitivity and 12% precision. In 60 patients, sensitivity achieved 100%. Low precision indicates that 88% of detections reflect the suspicious non-seizure activity that becomes a subject of manual sorting by an medical expert. Due to the rare nature of seizures, their number hardly surpasses 10 resulting in the overall number of detections below 100. Thus, running a seizure detection algorithm reduced the expert’s workload up to 95%.

Unlike the traditional classification algorithms, outlier detection incorporates the rare nature of seizures, hence handling the class imbalance problem. Classifying EEG data into two classes, “seizure” and “non-seizure”, one finds that the distribution of examples across these classes is heavily biased. Most of the classification algorithms use an assumption of an equal number of examples for each class; therefore, they demonstrate poor predictive performance for the minority class^46^. The popular methods to handle imbalance data rely on the oversampling of the minority class or undersampling of the majority class^47^. These methods (known as external methods) aim at balancing data and require advanced preprocessing procedures^48^. An alternative approach is employing the design of a new classification algorithm to tackle the bias produced by the imbalanced data (internal methods). A working solution is the one-class classifiers, which fit models using only a single class of data^49^. A one-class SVM^37–39^ is an example of one-class classifiers used for outlier detection^50^ including the detection of extreme events^51,52^.

Unlike the supervised algorithms, outlier detection methods may train and operate on the data of a single patient, hence handling the overfitting. The majority of existing seizure detection methods rely on supervised ML algorithms^53^ and face a high possibility of overfitting. The EEG data demonstrate high variability between patients and even between the different recordings of the same patient. Thus, there is a risk that the model fits specific features of the particular subject and fails on the data of other subjects. Addressing this issue requires forming highly representative training data, which is a challenging task in epileptology. Unsupervised algorithms use unlabeled data and perform its clusterization^21^. For seizure detection, they cluster EEG data in two clusters: “seizure” and “normal activity”^54^. For the one-class SVM, we tested how its performance depends on the training data and found the better performance when it classifies the data of the same subject rather than others. These results evidence that there are subject-specific epilepsy-related characteristics of EEG. Thus, we theorize that these individual features are more important for the classifier than the common ones derived from the dataset of multiple subjects.

**Table 3:** Results of data analysis with SVM classifier: workload reduction.

sub	tip, min	tFp, min	tbase, min	Preduc, %
2	1	24	480	94.79
4	2	11	480	97.29
5	2	6	480	98.33
6	4	9	480	97.29
7	2	36	480	92.08
8	2	83	480	82.29
9	5	20	480	94.79
10	2	22	467	94.86
12	7	58	5050	98.71
14	2	8	1246	99.20
16	3	8	440	97.50
17	2	90	480	80.83
18	2	20	480	95.42
19	2	55	480	88.13
20	1	40	480	91.46
21	5	73	2029	96.16
23	2	6	480	98.33
25	2	13	480	96.88
26	2	16	480	96.25
28	4	6	405	97.53
29	1	8	480	98.13
30	1	20	480	95.63
31	1	9	480	97.92
32	4	29	480	93.13
33	2	12	480	97.08
38	1	15	296	94.59
39	1	12	480	97.29
40	3	12	480	96.88
45	3	9	480	97.50
46	2	13	480	96.88
48	1	12	480	97.29
50	3	38	338	87.87
51	1	6	480	98.54
52	1	6	417	98.32
53	3	12	480	96.88
55	7	15	315	93.02
56	2	14	480	96.67
57	2	16	480	96.25
59	2	7	480	98.13
60	4	20	480	95.00
61	2	10	480	97.50
62	4	22	480	94.58
64	2	20	480	95.42
65	1	6	480	98.54
66	1	25	480	94.58
67	4	6	405	97.53
68	2	6	480	98.33
69	4	5	480	98.13
70	4	3	480	98.54
71	2	7	481	98.13
72	2	9	480	97.71
73	3	6	480	98.13
74	1	29	1813	98.35
76	6	12	1539	98.83
77	2	7	281	96.80
78	3	3	281	97.86
79	2	17	497	96.18
81	4	13	281	93.95
82	4	15	480	96.04
83	4	24	480	94.17
Mean				95.84
Std error				0.46

We feed the algorithm with the interpretable feature (EEG power in the 2–5 Hz frequency range) which demonstrates extreme behavior during seizures. Direct application of ML algorithm to the raw EEG data barely produces a reasonable output. Thus, it requires using informative input features, which are identified through the feature selection procedure. Informative feature space may be constructed from the data using deep-learning (DL) methods. Sometimes, DL enables achieving outstanding performance but it utilizes features that lack interpretation. In supervised algorithms, it increases the risk of overfitting since the model fits the data in the data-specific feature space. An alternative way is constructing features manually using the expert-level knowledge of the dataset. In the multichannel EEG data, the high-dimensional feature space can be derived by the time-frequency decomposition^55–57^. Many researchers proposed time-domain features, e.g., line length, frequency, and energy^58,59^. The repeatability, regularity (periodicity), synchronicity, and amplitude variation of EEG are also the major time-domain features differentiating seizure from the normal activity^60^. The most understandable way is using features whose significance is supported by the results of EEG research. For epilepsy, a bulk of studies report EEG biomarkers distinguishing seizures from the normal EEG^61,62^. In this study, we use the previous results proving extreme behavior of the 2–5 Hz power during seizures^15,16^.

We suppose that the interpretability gives us prior knowledge about the ability of an algorithm to handle the data of current patient, hence assessing its credibility. Comparing the data of the subjects from 100%-precision group with others, we observe distinct probability distributions. In the 100%-precision group, the probability distribution has a heavy tail indicating the presence of the extreme events. For the rest of the subjects, the probability of the extreme events is smaller and the tail is close to exponential. The detailed analysis reveals that the tail in the 100%-precision group almost solely consists of the detected seizures. For the rest of the subjects, the actual seizures belong to the 95% of data (see Fig. 5B).

Finally, we estimate a possible practical effect of using our algorithm in a decision-support system. The analysis of the whole dataset with a total duration of $t_{base}$ can be now substituted with the analysis of the subset with the duration of $t_{reduc}$. This subset includes all episodes marked as seizures by the algorithm: true positives (TPs) with a total duration of $t_{TP}$ and false positives (FPs) with $t_{FP}$, so the duration of the subset is $t_{reduc}=t_{TP}+t_{FP}$. We assume that the time required for the expert to process this data is proportional to the size of the dataset. Thus, the workload reduction percentage is $P_{reduc}=100\%(t_{base}-t_{reduc})/t_{base}$.

For the 100%-precision group, we found that the workload reduction percentage was $P_{reduc}=95.84 \pm 0.46$ (mean ± SE) (see Table 3). Thus, the expert will spend $\sim \,95\%$ less time detecting seizures, so the patient will earlier be provided with a confirmed diagnosis and get the necessary treatment. Moreover, the time reduction may prevent the fatigue of the expert, which occurs during the analysis of prolonged EEG datasets and increases the probability of misdiagnosis.

The principal limitation of the present study is that it did not aim to develop an optimal algorithm with characteristics (sensitivity and precision) superior to other methods for seizure detection or the results of a medical expert. Here, we demonstrate the concept of applying the outlier detection methods to the seizure selection problem, with the extreme event theory applied to the considered problem supporting the choice of this class of methods. This makes the developed approach interpretable, and one should consider the proposed method as the basis of a medical decision support system. It is important to note that the developed method can greatly facilitate the work of the expert, reducing the duration of the analyzed dataset up to 95%. Also, note that we chose one-class SVM in our work because this classifier is the traditional algorithm for solving the problem of detecting outliers. Of course, SVM can be replaced by other better methods. Conducting such research is intriguing and planned for our future studies.

## Conclusions

Based on extreme event theory, we propose using an outlier detection algorithm, one-class SVM, to detect epileptic seizures on the human EEG using the 2–5 Hz power as a feature. In most patients, the probability distribution of the 2–5 Hz EEG power was heavy-tailed, demonstrating the presence of extreme events. For them, one-class SVM achieved 100% sensitivity and 12% precision. Low precision indicates that 88% of detections reflect the suspicious non-seizure activity that becomes a subject of manual sorting by an expert. Due to the rare nature of seizures, their number hardly surpasses 10, resulting in the overall number of detections below 100. Thus, running a seizure detection algorithm reduced the expert’s workload up to 95%. For the rest of the subjects, the probability distribution barely displayed the manifestations of extreme behavior, and SVM demonstrated a poor ability to detect seizures. Finally, we showed that one-class SVM used a single subject’s data for training; therefore, it was stable against between-subject variability. Our results demonstrate an effective convergence between the extreme value theory, a physical concept, and the outlier detection algorithms, a machine learning concept, toward solving the meaningful task of medicine.

## Data Availability

The data that support the findings of this study are available from National Medical and Surgical Center named after N. I. Pirogov of Russian Healthcare Ministry but restrictions apply to the availability of these data, which were used under license for the current study, and so are not publicly available. Data are however available from the authors upon reasonable request and with permission of National Medical and Surgical Center named after N. I. Pirogov of Russian Healthcare Ministry.
